# Macular hole formation and spontaneous closure after vitrectomy for vitreomacular traction documented in spectral-domain optical coherence tomography

**DOI:** 10.1186/1471-2415-14-17

**Published:** 2014-02-19

**Authors:** Dominik Odrobina, Iwona Laudańska-Olszewska, Piotr Gozdek, Mariusz Maroszyński, Michael Amon

**Affiliations:** 1 Ophthalmology Clinic of St. John Boni Frates Lodziensis, ul. Kosynierów Gdyńskich 17, Al. Hippiczna, Lodz, Poland; 2 Academic Teaching Hospital of St. John, Johannes von Gott Platz 1, Vienna 1020, Austria

**Keywords:** Vitreomacular traction, Full-thickness macular hole, Spectral-domain optical coherence tomography

## Abstract

**Background:**

We present a case of a macular hole formation and its spontaneous closure after vitrectomy for vitreomacular traction. To our knowledge, it is the first description of spontaneous closure of the macular hole after vitrectomy for vitreomacular traction.

**Case presentation:**

A 78-year-old woman presented decreased visual acuity and metamorphopsia in the right eye due to vitreomacular traction. A vitrectomy with internal limiting membrane peeling and an air tamponade was performed in the right eye. Spectral-domain optical coherence tomography was obtained during all visits.

Seven days after the vitrectomy, the spectral-domain optical coherence tomography showed a resolved vitreomacular traction and a full-thickness macular hole. Examination after a further three weeks showed that the full-thickness macular hole had spontaneously closed. 5 months later spectral-domain optical coherence tomography showed a normal foveal contour without intraretinal microcystic spaces and a resolution of the photoreceptor and external limiting membrane elevation.

**Conclusions:**

While performing a vitrectomy for vitreomacular traction posterior hyaloid membrane creates anterior-posterior traction on the fovea, and, during detachment, retinal layer damage occurs in the macular area and a full-thickness macular hole may develop. Removal of the anterio-posterior vitreous traction may play the main role and may help the spontaneous closure of the macular hole after vitrectomy for vitreomacular traction.

## Background

Pars plana vitrectomy is a well-established surgical procedure for the treatment of vitreomacular traction (VMT). Despite the high percentage of anatomic successes, some postoperative complications may occur, such as a macular hole [1-3].

Although the pathogenesis of macular hole formation after vitrectomy for VMT is not fully understood, new diagnostics methods such as Spectral-domain optical coherence tomography (SD-OCT) have provided additional information about this process.

To our knowledge, the current case is the first description of the macular hole formation and spontaneous closure after vitrectomy for VMT clearly documented step by step in SD-OCT (Spectralis; Heidelberg Engineering, Heidelberg, Germany).

## Case presentation

**Figure 1:**
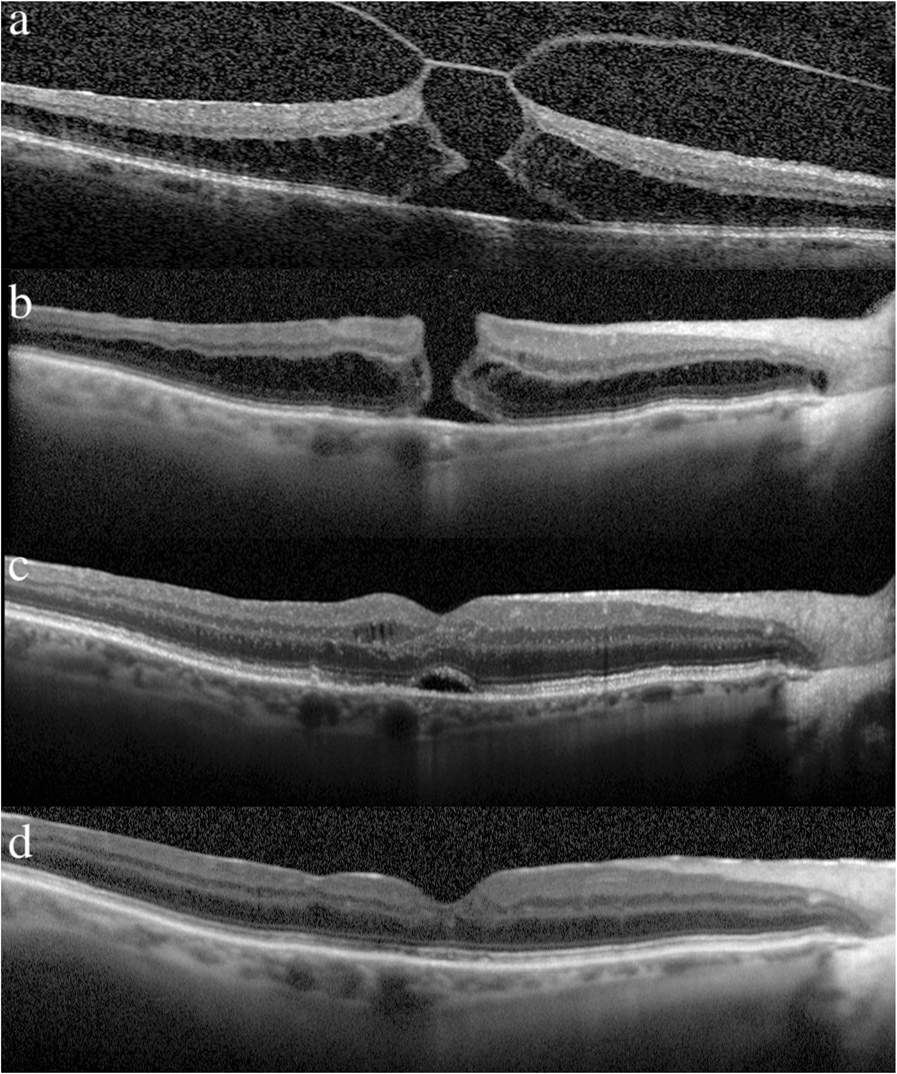
**Spectral-optical coherence tomography before and after vitrectomy for vitreomacular traction. a**: A 78-year-old female presented with visual acuity 0.04 and metamorphopsia. SD-OCT examination showed the vitreomacular traction (VMT) with an outer lamellar macular hole and an abnormal foveal contour. **b**: Seven days after the vitrectomy with ILM peeling visual acuity increased to 0.08. SD-OCT showed the full-thickness macular hole (FTMH) with intraretinal cystoid spaces on the both edges of the hole. **c**: One month later the patient presented with increased of visual acuity to 0.2. SD-OCT clearly documented closed macular hole with an elevation of the photoreceptor layer and of the external limiting membrane (ELM) in the fovea region and intraretinal microcystoid spaces. **d**: 5 months later visual acuity improved to 0.5. SD-OCT examination showed normal fovea contour without subretinal fluid with no evidence of space in the outer layers of the retina.

A 78-year-old female presented a visual acuity of 0,04 and metamorphopsia in the right eye that had lasted for 6 months after an uncomplicated phacoemulsification with lens implantation performed in another department. SD-OCT examination showed VMT with an outer lamellar macular hole and an abnormal foveal contour (Figure 1a). A vitrectomy with internal limiting membrane (ILM) peeling and an air tamponade was performed by the author (D.O.). After the complete three-port pars plana vitrectomy, 0.15% trypan blue solution (Membrane Blue Dual-Dorc, Zuidland, The Netherlands) was injected for 60 seconds. After removal of the trypan blue, ILM peeling was performed in the macular area. At the end of the surgery, a fluid-air exchange was performed. Patient received non-supine positioning (NSP) for 5 postoperative days.

Seven days after the vitrectomy, the SD-OCT (Figure 1b) showed a resolved VMT and full-thickness macular hole (FTMH) with cystoid spaces on the edges, as reported in the literature [3]. After a further three weeks, SD-OCT (Figure 1c) showed that the FTMH had spontaneously closed. The image shows a normal foveal contour with an elevation of the photoreceptor layer and of the external limiting membrane (ELM) in the fovea region and intraretinal microcystoid spaces.

Figure 1d was recorded with SD-OCT 5 months later and showed a normal foveal contour without intreretinal microcystic spaces and a resolution of the photoreceptor and ELM elevation.

## Conclusions

The posterior hyaloid membrane may play the main role in forming the FTMH in VMT. The posterior hyaloid membrane creates anterio-posterior traction on the fovea, and, during detachment, retinal layer damage occurs in the macular area and FTMH may develop. In our patient, the posterior hyaloid membrane is still attached to the ILM, which is the reason that FTMH did not appear. Anterio-posterior traction acts as a conglomeration of these two tissues. This traction was pulled up to the retina, so that the edges of the hole were elevated high, but it was stabilized by the ILM and the posterior hyaloid membrane. The posterior hyaloid membrane creates traction on the ILM, and, during surgically induced detachment, the complex of the posterior hyaloid membrane and ILM was probably removed in the macular area and FTMH develops. As suggested by Charles [4], when operating on VMT cases, the posterior vitreous cortex should be delaminated from the fovea prior to any removal of the vitreous to prevent tearing the fovea. FTMH may develop in its natural course and after vitrectomy for VMT [3,5]. In none of these cases did spontaneous closure of the FTHM develop; they required another surgery to close the macular hole.

Eckardt et al. showed that about 91% of macular holes closed 3 days after surgery [6]. Also Jumper et al. presented that macular holes with a diameter < 400 μm were closed 1 day after surgery [7]. Some authors reported that in non-supine positioning (NSP) patients, about 90% of macular holes were closed [8]. This is why after vitrectomy the patient received postoperative NSP only for 5 days. The necessity of face-down positioning (FDP) after vitrectomy with air/gas tamponade for macular hole surgery is still unclear. In our patient, after air absorption seven days after vitrectomy the macular hole remained open [Figure 1b] and closed later [Figure 1c]. Based on this case, we can see that neither the air tampoande nor the position after vitrectomy affected the closure of the hole.

In our opinion, there are two possible mechanisms that could cause spontaneous closure of the macular hole. After surgically inducing posterior hyaloid detachment, the edges of the hole were left at the bottom by the resolution of the traction. This could cause a decrease in the distance between the edges of the hole, and put them together by reducing intraretinal cystoid spaces. The release of the mechanical traction may be the main reason for the eventual closure of the macular hole. On the other hand, ILM peeling induces glial cell proliferation across the hole and this mechanism may also help the spontaneous closure of macular hole.

While performing vitrectomy for VMT, the posterior hyaloid membrane creates anterior-posterior traction on the fovea, and, during detachment, retinal layer damage occurs in the macular area and FTMH may develop. Removal of the anterio-posterior vitreous traction may play the main role and may help the spontaneous closure of the macular hole after vitrectomy for vitreomacular traction. ILM peeling may also help the spontaneous closure of a macular hole.

## Consent

Written informed consent was obtained from the patient for publication of this Case report and any accompanying images. A copy of the written consent is available for review by the Editor of this journal.

## Abbreviations

SD-OCT: Spectral-domain optical coherence tomography; VMT: Vitreomacular traction; FTMH: Full-thickness macular hole; ILM: Internal limiting membrane; ELM: External limiting membrane.

## Competing interests

This research was not supported by any competing interests or grants.

## Authors’ contributions

DO data collection performing surgery drafting of manuscript analysing and interpreting the data final approval of manuscript. ILO: analysing and interpreting the data final approval of manuscript. PG: final approval of manuscript. MM: final approval of manuscript. MA: final approval of manuscript. All authors read and approved the final manuscript.

## Pre-publication history

The pre-publication history for this paper can be accessed here:

http://www.biomedcentral.com/1471-2415/14/17/prepub
